# Differential adiponectin signalling couples ER stress with lipid metabolism to modulate ageing in *C. elegans*

**DOI:** 10.1038/s41598-017-05276-2

**Published:** 2017-07-11

**Authors:** Emmanouil Kyriakakis, Nikolaos Charmpilas, Nektarios Tavernarakis

**Affiliations:** 1 Institute of Molecular Biology and Biotechnology, Foundation for Research and Technology - Hellas, Nikolaou Plastira 100, Heraklion 70013, Crete, Greece; 2 Department of Biology, University of Crete, Heraklion 70013, Crete, Greece; 3 Department of Basic Sciences, Faculty of Medicine, University of Crete, Heraklion 71110, Crete, Greece

## Abstract

The metabolic and endocrine functions of adipose tissue and the ability of organisms to cope with cellular stress have a direct impact on physiological ageing and the aetiology of various diseases such as obesity-related pathologies and cancer. The endocrine effects of adipose tissue are mediated by secreted adipokines, which modulate metabolic processes and influence related maladies. Although a plethora of molecules and signaling pathways associate ageing with proteotoxic stress and cellular metabolism, our understanding of how these pathways interconnect to coordinate organismal physiology remains limited. We dissected the mechanisms linking adiponectin signalling pathways and endoplasmic reticulum (ER) proteotoxic stress responses that individually or synergistically affect longevity in *C. elegans*. Animals deficient for the adiponectin receptor PAQR-1 respond to ER stress, by rapidly activating the canonical ER unfolded protein response (UPR^ER^) pathway, which is primed in these animals under physiological conditions by specific stress defence transcription factors. PAQR-1 loss enhances survival and promotes longevity under ER stress and reduced insulin/IGF-1 signalling. PAQR-1 engages UPR^ER^, autophagy and lipase activity to modulate lipid metabolism during ageing. Our findings demonstrate that moderating adiponectin receptor -1 activity extends lifespan under stress, and directly implicate adiponectin signalling as a coupler between proteostasis and lipid metabolism during ageing.

## Introduction

Ageing is characterized by deterioration of physiological regulatory functions, and progresses towards frailty and age-associated pathology. Cellular adaptation to extrinsic and intrinsic stress stimuli relies on a wide range of cellular processes that are tightly controlled. Failure of organisms to quickly and efficiently respond to stress has a direct negative impact on homeostasis, leading to feeble healthspan and reduced survival^1–4^. Although a large number of stress responses have been associated with ageing, the intersecting regulatory networks coordinating these responses remain enigmatic.

Perturbed protein homeostasis (proteostasis) is one of the hallmarks of ageing^1, 3, 5, 6^. Several compensatory stress response mechanisms, acting at the cellular or organelle level, function to preserve the stability and functionality of the proteome^1, 2^. The heat shock response (HSR) is one of the best characterized responses aimed at maintaining cytoplasmic proteome integrity^2, 5^. Organelle-specific response pathways are regulated by the endoplasmic reticulum (ER) unfolded protein response (UPR^ER^), the less well characterized mitochondrial unfolded protein response (UPR^mt^), the peroxisomal quality control system and autophagy^2, 7, 8^. Deterioration of these homeostatic mechanisms is a general feature of ageing and also occurs in several age-associated diseases. However, it remains unclear whether deterioration is simply a consequence of the ageing process or a significant causative factor.

Perturbations of ER proteostasis results in ER stress and initiation of the UPR^ER^ aimed at ameliorating accumulation of unfolded proteins. ER stress and impaired UPR^ER^ signalling have been implicated in the aetiology of age-related maladies including metabolic diseases^9^. ER stress aggravates metabolic dysfunction, thereby contributing to enhanced lipogenesis, obesity and insulin resistance. In a reciprocal manner, obesity, caused by imbalance between energy intake and expenditure, induces ER stress^10^. Thus, a bidirectional relationship between ER-mediated proteotoxicity and disturbed cellular metabolism exists. However, molecular components mediating the intersection between ER proteostasis and metabolic homeostasis, and their impact on healthy ageing are poorly understood.

Adipose tissue functions primarily as a reserve of energy in the form of triglycerides. However, it is now recognized that adipose tissue is also a highly active metabolic and endocrine organ, and that secreted adipose-derived hormones (adipokines) are important for metabolic homeostasis^11, 12^. Adipokines exert their effect systemically, contributing to whole body metabolic homeostasis. The adipokine adiponectin is particularly involved in this regulatory axis because of its insulin sensitizing property and role in energy homeostasis^13, 14^. Adiponectin, together with cognate adiponectin receptors which mediate its anti-diabetic and anti-atherogenic metabolic actions, are potential versatile therapeutic targets for obesity-related diseases and the metabolic syndrome.

We studied the role of adiponectin signalling via specific adiponectin receptors^15^, in *C. elegans* ageing and metabolic homeostasis. Our findings reveal that adiponectin signalling differentially modulates proteotoxic stress responses and lipid metabolism to influence stress resistance and ageing.

## Results

### The *C. elegans* adiponectin receptors differentially influence resistance to ER Stress and animal survival

**Figure 1:**
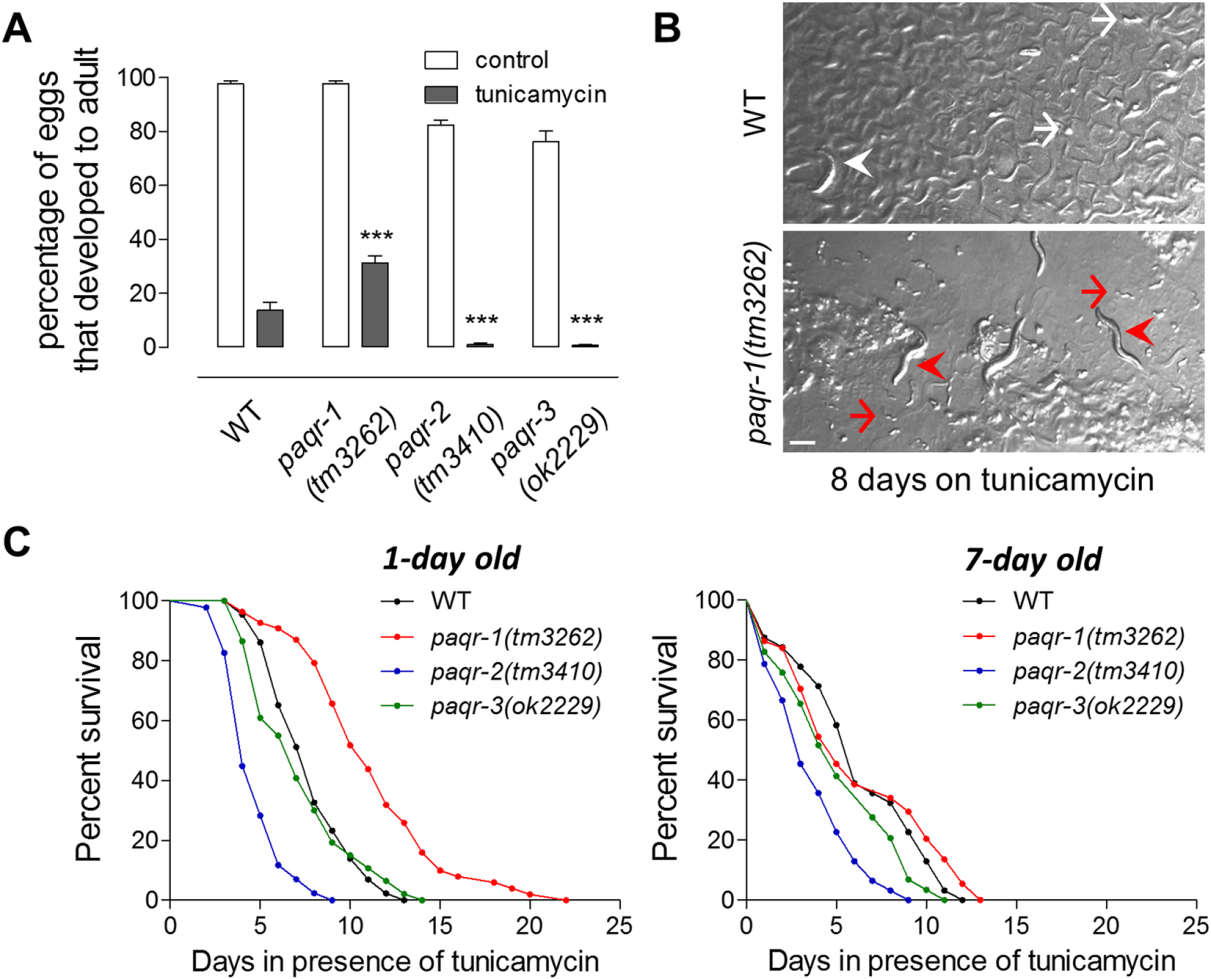
PAQR-1 depletion augments survival of ER stressed animals. (**A**) Eggs derived from WT, *paqr-1(tm3262)*, *paqr-2(tm3410)* and *paqr-3(ok2229)* strains were laid on plates in the absence (white bars) or presence of 5 μg/ml tunicamycin (black bars). Percentage of eggs that developed into mature adults was scored. Each strain was scored on four independent plates and each experiment was repeated independently at least three times. Error bars represent SEM for repeat plates within the experiment. ***P < 0.001 *vs*. WT + tunicamycin. (**B**) Survival of eggs that developed into mature adults after 8 days on tunicamycin (5 μg/ml). Representative images illustrating survival of WT and *paqr-1(tm3262)* strains are shown. Differences on bacterial lawn thickness at the time of observation indicate changes in mobility and viability. Scale bar, 250 μm. White arrowhead indicates a dead worm; white arrows indicate eggs that never developed into mature adults; red arrowheads indicate live worms; red arrows indicate newly hatched eggs. (**C**) WT (black), *paqr-1(tm3262)* (red), *paqr-2(tm3410)* (blue), *and paqr-3(ok2229)* (green) animals were transferred at day 1 or day 7 onto plates containing 50 μg/ml tunicamycin and survival was monitored. Lifespan values are given in Table S1.

To investigate the impact of the *C. elegans* adiponectin receptors PAQR-1, PAQR-2 and PAQR-3 on the sensitivity to ER stress, we exposed wild-type (WT) and *paqr-1(tm3262), paqr-2(tm3410)* and *paqr-3(ok2229)* mutant animals to the ER stress inducers tunicamycin and thapsigargin, which inhibit N-linked glycosylation and sarco/endoplasmic reticulum Ca^2+^-ATPase (SERCA), respectively. Compared to WT animals, *paqr-1* mutants were more resistant, whereas *paqr-2* and *paqr-3* mutants were more sensitive, to conditions of ER stress (Fig. 1A and Supplementary Fig. S1). *paqr-1* silencing also increases ER stress resistance (Supplementary Fig. S2A), however the relative improvement is weaker than the one found in *paqr-1* mutant animals. This is likely due to partial loss of PAQR-1 by RNAi. Next, and to determine whether adiponectin receptors also impact general proteostatic mechanisms initiated by HSR, WT and mutant animals were exposed to non-permissive temperatures. No significant differences in survival between the different mutants under heat stress conditions were observed (Supplementary Fig. S3), suggesting that the observed effects are specific to the ER stress response. Notably, and despite the fact that exposure to ER stress, from early in development, was fatal for most animals and their progeny, animals lacking a functional PAQR-1 exhibited substantial adaptation to adverse conditions. We observed that the small percentage of *paqr-1* mutants that developed into adults during the ER stress test remained viable more than 8 days after hatching on plates containing tunicamycin, whereas WT animals, *paqr-2* or *paqr-3* mutants that reached adulthood under the same conditions perished in less than 3 days. Moreover, the *paqr-1* mutants that survived under harsh experimental conditions, in contrast to WT, *paqr-2* or *paqr-3* mutant animals, were able to lay viable progeny; however, the progeny never reached adulthood (Fig. 1B and Supplementary Fig. S4, images for *paqr-1* and WT shown).

Under physiological conditions, lifespan is not significantly affected by the *paqr-1(tm3262)* or *paqr-3(ok2229)* mutations, whereas it is reduced by the *paqr-2(tm3410)* mutation^15^. We could recapitulate these findings (Supplementary Fig. S5). However, since *paqr-1(tm3262)* animals are more resistant to ER stress, we queried whether their survival under conditions of ER stress is improved. Lifespan assays performed following challenging 1-day old animals with tunicamycin showed that indeed *paqr-1(tm3262)* animals survived substantially longer than the WT animals, or *paqr-2(tm3410)* and *paqr-3(ok2229)* mutants (Fig. 1C left panel). In a similar setting WT animals subjected to *paqr-1(RNAi)* survived also substantially better than the control (EV) animals (Supplementary Fig. S2B left panel). WT and *paqr-3(ok2229)* animals showed comparable sensitivity, whereas *paqr-2(tm3410)* animals were less resistant (Fig. 1C left panel). Interestingly, when 7-day old animals were challenged with tunicamycin the survival advantage of *paqr-1(tm3262)* animals or WT animals subjected to *paqr-1(RNAi)* was no longer evident (Fig. 1C right panel and Supplementary Fig. S2B right panel). Given that the ability to activate the UPR^ER^ and initiate protective mechanisms against ER stress declines with age^16, 17^, our findings indicate that UPR^ER^ activity is important for the survival advantage of *paqr-1(tm3262)* mutants under ER stress.

### PAQR-1 mitigates resistance to ER stress by impeding the canonical UPR^ER^ pathway

**Figure 2:**
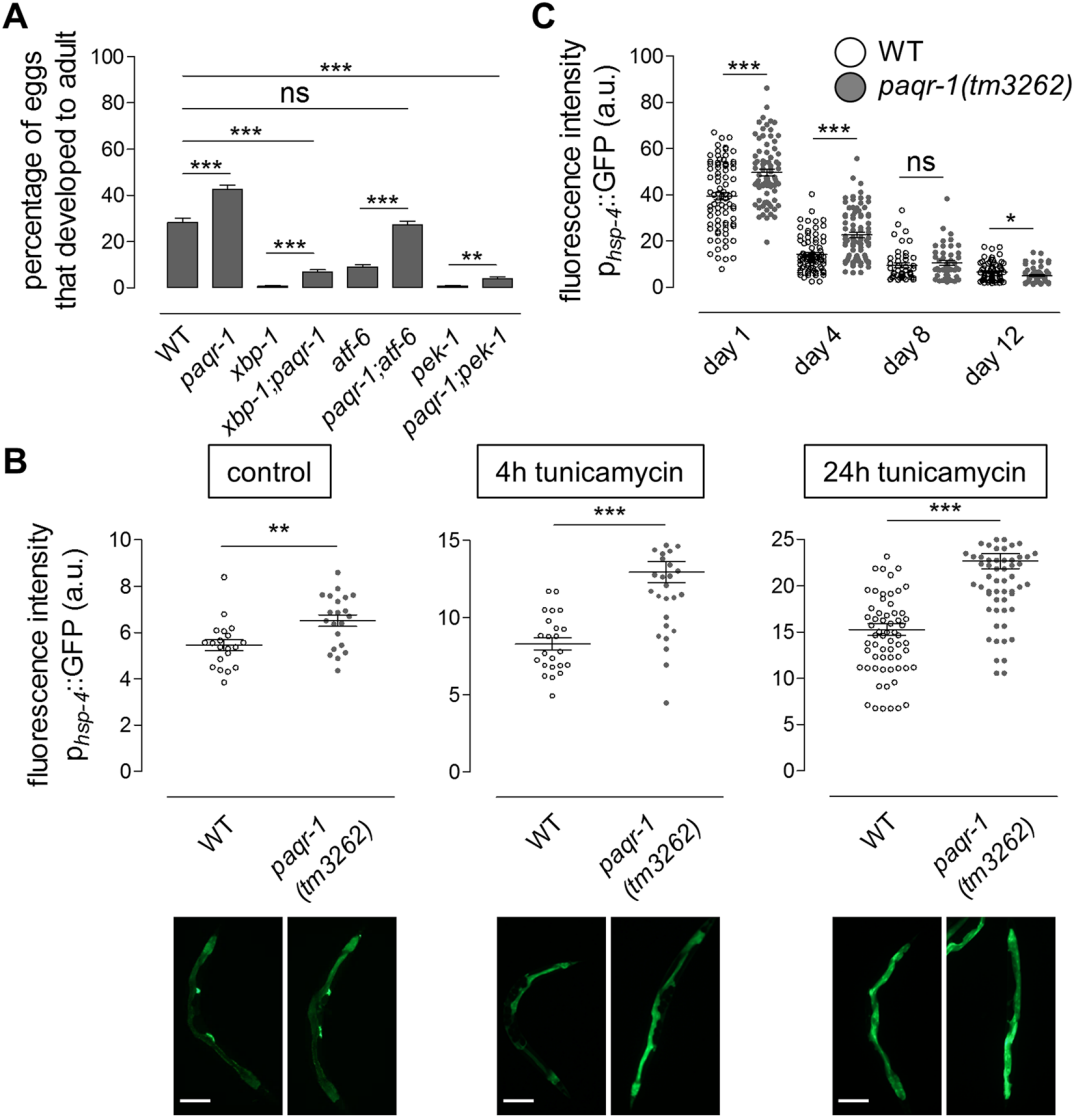
PAQR-1 regulates UPR^ER^ activity through XBP-1 and PEK-1. (**A**) Eggs from WT or *paqr-1(tm3262)* animals containing *xbp-1(tm2457), pek-1(ok275)* or *atf-6(ok551)* mutations were laid on plates in the presence of 5 μg/ml tunicamycin. Percentage of eggs that developed into mature adults was scored. Each strain was scored on four independent plates and each experiment was repeated independently at least three times. Error bars represent SEM for repeat plates within the experiment. (**B**) Mean fluorescence intensity values of day 4 WT (white) or *paqr-1* mutant (grey) animals carrying the p_*hsp-4*_GFP transgene, without (control) or with tunicamycin treatment (5 μg/ml tunicamycin for 4 or 24 h). Representative images are shown. Scale bar, 100 μm. (**C**) Quantification of WT (white) or *paqr-1* mutant (grey) animals carrying the p_*hsp-4*_GFP transgene, treated with tunicamycin (5 μg/ml, 24 h) at the indicated ages. Each experiment was repeated independently three times. Values in histograms represent means ± SEM. *P < 0.05; **P < 0.01; ***P < 0.001.

The evolutionary conserved IRE-1/XBP-1 pathway is mainly orchestrating the transcriptional response to ER stress in *C. elegans*. In addition, two other branches of UPR^ER^, are mediated by PERK-1/PEK-1 and ATF-6. To investigate whether activation of the canonical UPR^ER^ pathway is required for increased ER stress resistance upon PAQR-1 depletion, we examined ER stress-sensitive *xbp-1, pek-1* and *atf-6* mutants that also carry the *paqr-1(tm3262)* lesion. We found that loss of PAQR-1 fully restored ER stress resistance in *atf-6* mutants and slightly increased ER stress resistance of *xbp-1* and *pek-1* mutants (Fig. 2A). A two-way ANOVA analysis showed that *paqr-1* plays a significant role on its own, which however, is highly dependent on the genetic background. These findings suggest a synergistic effect mediated at least by two arms of the UPR response. Upon ER stress, transcription of *hsp-4* (homolog of mammalian chaperone BiP/Grp78) is upregulated in an IRE-1/XBP-1 dependent manner. Therefore, we additionally examined UPR^ER^ activation in *paqr-1(tm3262)* mutants using a p_*hsp-4*_GFP reporter for UPR^ER^ activation. Basal activation of *hsp-4* was induced by PAQR-1 deficiency (Fig. 2B left panel). Exposure of wild-type animals to tunicamycin for either 4h or 24h induced *hsp-4* expression and this response was amplified in *paqr-1(tm3262)* mutants, or WT animals subjected to *paqr-1(RNAi)* (Fig. 2B middle and right panels and Supplementary Fig. S2C).

In *C. elegans*, ER stress responses decline early during adulthood and are almost abolished by the age of 7 days^17^. We queried whether loss of PAQR-1 confers enhanced UPR^ER^ during ageing. Tunicamycin treatment of wild-type animals at ages ranging from day 1 to day 12 of adulthood potently induced *hsp-4* expression early during adulthood, while induction markedly declined with age (Fig. 2C). PAQR-1 depletion amplified tunicamycin-triggered UPR^ER^ as manifested by monitoring *hsp-4* expression (Fig. 2C).

We finally examined whether differential activation of UPR^ER^ by tunicamycin is due to alterations in either uptake or intestinal release of the stressor. Assessment of ultradian rhythms (pharyngeal pumping, defecation), with or without tunicamycin, showed no differences between WT and *paqr-1(tm3262)* mutant animals (Supplementary Fig. S6). Collectively, our findings indicate that loss of PAQR-1 function potentiates ER stress responses. Animals bearing *paqr-1* lesions mount a rapid and enhanced response to ER stress, revealing a novel role for PAQR-1 in fine-tuning the canonical UPR^ER^ pathway.

### PAQR-1 depletion abrogates the effects of low insulin/IGF-1 signalling on survival and stress resistance

**Figure 3:**
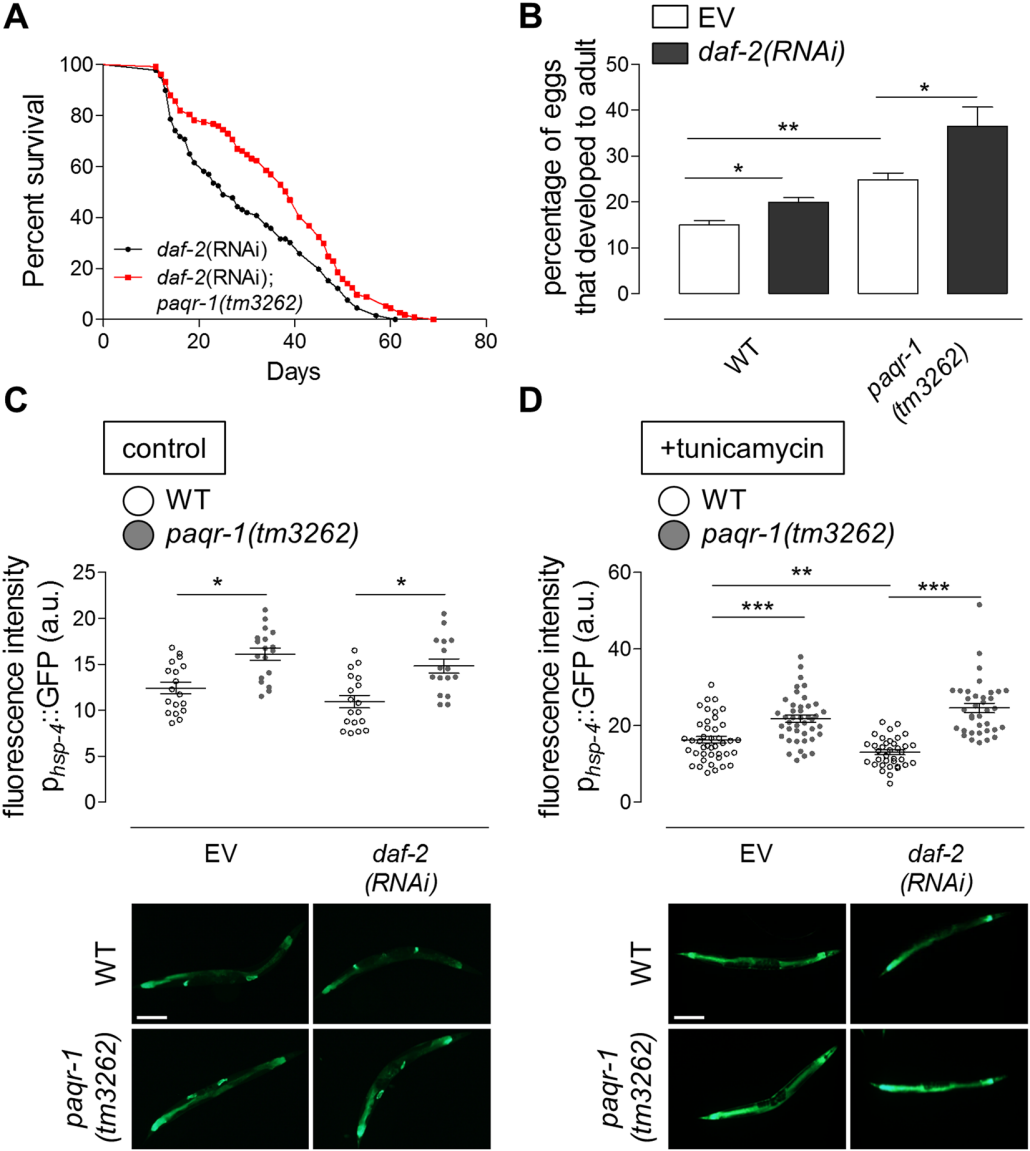
PAQR-1 deficiency extends the lifespan of *daf-2* mutants and induces their survival during ER stress. (**A**) Survival was monitored under normal conditions. Mutant *paqr-1(tm3262)* further extends the lifespan of long-lived, insulin signalling-defective *daf-2*(*RNAi*) animals. Lifespan values are given in Table S1. (B) Eggs from WT or *paqr-1(tm3262)* mutant animals were laid on plates in the presence of 5 μg/ml tunicamycin and EV (white) or *daf-2(RNAi)* (black). Percentage of eggs that developed into mature adults was scored. (**C**) Basal mean fluorescence intensity values of day 4 WT (white) or *paqr-1* mutant (grey) animals carrying the p_*hsp-4*_GFP transgene. Representative images are shown. Scale bar, 100 μm. (**D**) Mean fluorescence intensity values of tunicamycin-treated (5 μg/ml, 24 h), day 4 WT (white) or *paqr-1* mutant (grey) animals carrying the p_*hsp-4*_GFP transgene. Representative images are shown. Scale bar, 100 μm. Values in histograms represent means ± SEM from experiments performed on at least three separate occasions. *P < 0.05; **P < 0.01; ***P < 0.001.

Reduced insulin/IGF-1 signalling increases resistance to a variety of insults and stressors, including heat, hypoxia, paraquat, DNA damaging agents, pathogens, tunicamycin and dithiothreitol (DTT), in *C. elegans* and other metazoans^18, 19^. Impairment of the insulin/IGF-1 signalling pathway also extends lifespan by promoting proteostasis, among others^18^. ER stress response pathways are important mediators of longevity in *C. elegans* DAF-2 (insulin/IGF-1 receptor) deficient animals. The IRE-1/XBP-1 branch of the UPR^ER^ pathway increases longevity and ER stress resistance of *daf-2* mutants by setting a lower UPR^ER^ threshold, through mechanisms that involve activation of the DAF-16/FOXO transcription factor^18^. Given that PAQR-1 is a negative regulator of UPR^ER^, we examined the effects of that *paqr-1* knockdown on the lifespan and ER stress resistance of animals with reduced insulin/IGF-1 signalling. We find that *paqr-1(tm3262)* mutants subjected to *daf-2* RNAi display enhanced survival compared to WT animals (Fig. 3A). *daf-2* silencing increases ER stress resistance in both WT animals and *paqr-1(tm3262)* mutants (Fig. 3B). *paqr-1* expression is not altered during ageing or under ER stress (Supplementary Fig. S7). The relative improvement in ER stress resistance upon *daf-2* RNAi silencing (≈ 1.6-fold above vector control) is weaker than that reported for *daf-2* mutant animals (≈ 4-fold above WT)^18^. This is likely due to partial inactivation of *daf-2* by RNAi. We find that PAQR-1 deficiency restores UPR^ER^ as manifested by HSP-4 expression in *daf-2(RNAi)* animals, and further induces HSP-4 expression both, under control and ER stress conditions (Fig. 3C, D). Notably, the effects of DAF-2 deficiency on UPR^ER^ induction were fully bypassed by PAQR-1 loss.

### Stress defence transcription factors coordinatedly mediate signalling via PAQR-1

**Figure 4:**
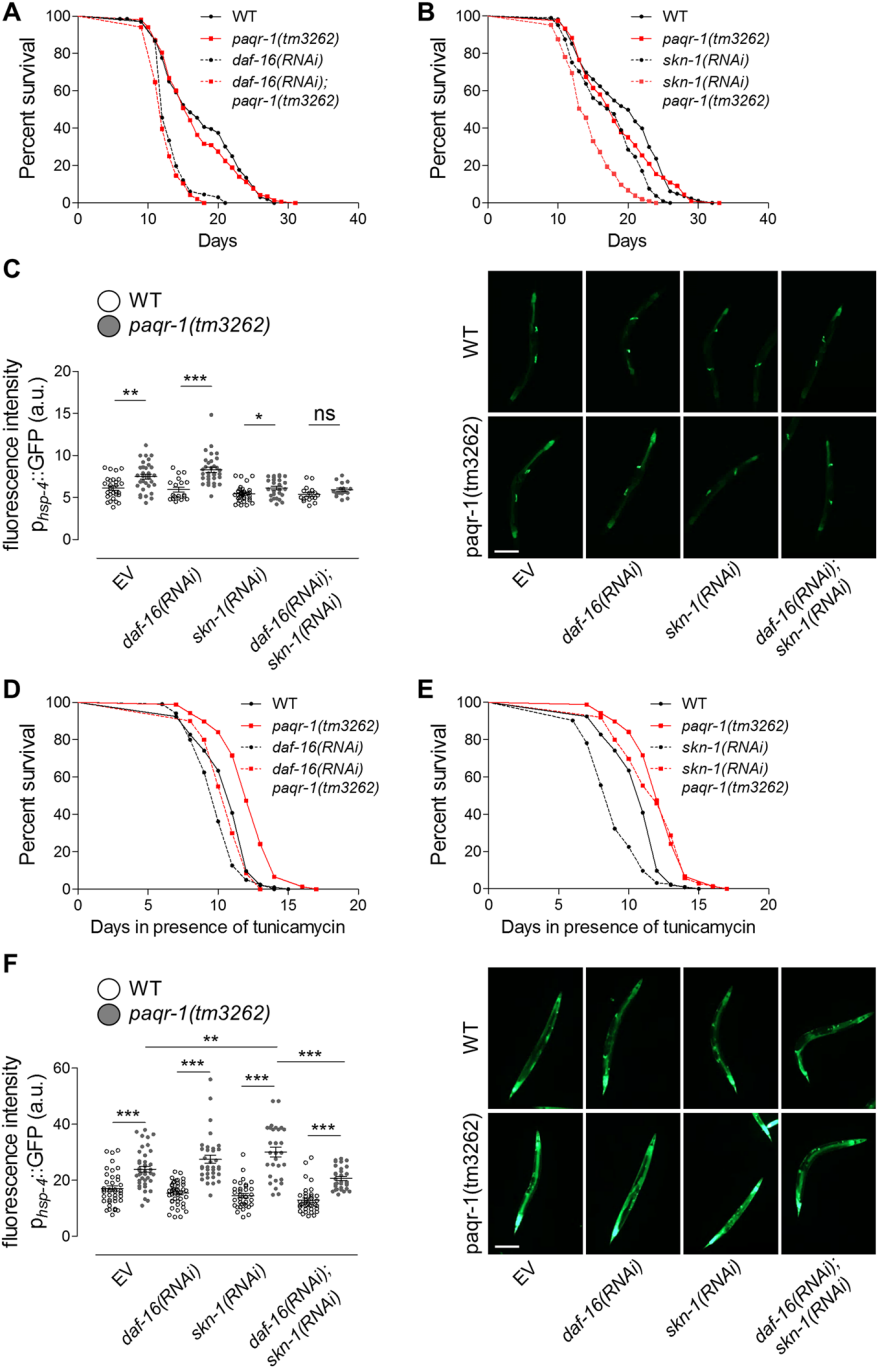
Involvement of SKN-1 and DAF-16 in PAQR-1-dependent effects on survival and UPR^ER^ activation. (**A**) Survival curves of WT (black) and *paqr-1(tm3262)* mutant (red) animals subjected to *daf-16(RNAi)* (dashed) under normal conditions. Lifespan values are given in Table S1. (**B**) Survival curves of WT (black) and *paqr-1(tm3262)* mutant (red) animals in the presence of *skn-1(RNAi)* (dashed) under normal conditions. Lifespan values are given in Table S1. (**C**) Mean fluorescence intensity values of day 4 p_*hsp-4*_GFP transgenic animals (WT (white) or *paqr-1* mutant (grey) background) subjected to *daf-16(RNAi)* and/or *skn-1(RNAi)*. Representative images are shown. Scale bar, 100 μm. (**D**) Survival curves of WT (black) and *paqr-1(tm3262)* mutant (red) animals subjected to *daf-16(RNAi)* (dashed) under ER stress (50 μg/ml tunicamycin). Lifespan values are given in Table S1. (**E**) Survival curves of WT (black) and *paqr-1(tm3262)* mutant (red) animals subjected to *skn-1(RNAi)* (dashed) under ER stress (tunicamycin 50 μg/ml). Lifespan values are given in Table S1. (**F**) Mean fluorescence intensity of tunicamycin-treated (5 μg/ml, 24h) day 4 p_*hsp-4*_GFP transgenic animals (WT (white) or *paqr-1* mutant (grey) background) subjected to *daf-16(RNAi)* and/or *skn-1(RNAi)*. Representative images are shown. Scale bar, 100 μm. Values in histograms represent means ± SEM from experiments performed on at least three separate occasions. *P < 0.05; **P < 0.01; ***P < 0.001.

The stress defence transcription factors DAF-16/FOXO and SKN-1/Nrf play pivotal roles downstream of the DAF-2 (insulin/IGF-1 receptor) to regulate longevity in *C. elegans*. These transcription regulators coordinate the expression of large sets of genes that influence longevity by promoting resistance to various stressors^18, 20–22^. We asked whether DAF-16 and/or SKN-1 mediate the enhancement of ER stress resistance upon PAQR-1 depletion. Under normal growth conditions, DAF-16 silencing shortened the lifespans of WT animals and *paqr-1(tm3262)* mutants to the same extent (Fig. 4A). SKN-1 silencing also shortened lifespan in both genetic backgrounds, with a more pronounced impact on *paqr-1(tm3262)* mutants (Fig. 4B). By using a *p*_*hsp-4*_GFP reporter, we found that that neither *skn-1*(*RNAi*) nor *daf-16(RNAi)* affected UPR^ER^ activation in WT animals under normal conditions (Fig. 4C and Supplementary Fig. S8). However, in *paqr-1(tm3262)* mutants, *daf-16(RNAi)* increased, and *skn-1(RNAi)* decreased expression of *hsp-4* induced by *paqr-1* lesion (Fig. 4C). Simultaneous silencing of *skn-1* and *daf-16* completely abrogated stimulatory effects of PAQR-1 depletion on *hsp-4* expression (Fig. 4C). The dependence of *hsp-4* upregulation by PAQR-1 deficiency on SKN-1 indicates that SKN-1 is a mediator of UPR^ER^ activation in *paqr-1(tm3262)* animals under normal growth conditions.

Under conditions of tunicamycin-induced ER stress, *daf-16(RNAi)* reduced survival of both WT animals and *paqr-1(tm3262)* mutants (Fig. 4D), while *skn-1*(*RNAi*) markedly reduced survival of WT animals, with no effect on *paqr-1(tm3262)* mutants (Fig. 4E). This suggests that augmented survival during ER stress in *paqr-1(tm3262)-*mutants is not mediated by SKN-1. UPR^ER^ activation under ER stress, as monitored by a p_*hsp-4*_GFP reporter, was not altered upon DAF-16 and/or SKN-1 depletion in WT animals. By contrast, *hsp-4* expression was significantly increased by *skn-1*(*RNAi*) and reduced by simultaneous DAF-16 and/or SKN-1 depletion in *paqr-1(tm3262)* mutants (Fig. 4F and Supplementary Fig. S8). Together, these findings indicate that SKN-1 is capable of regulating PAQR-1-dependent ER homeostasis, although its relative contribution depend on the state of stress experienced by the organism.

### PAQR-1 regulates lipid droplet turnover during ageing in ER-stressed animals

**Figure 5:**
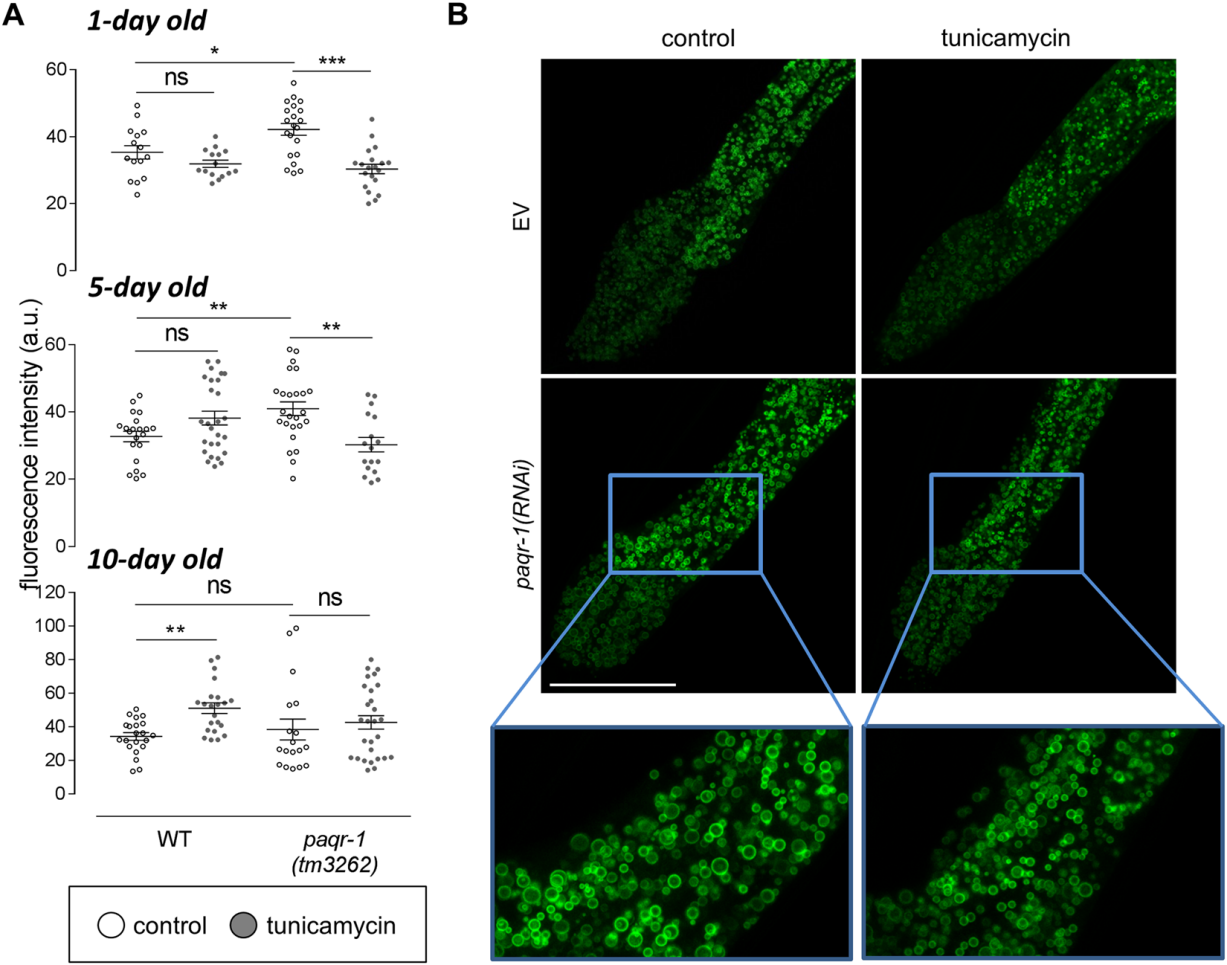
Lipid metabolism requires PAQR-1 during ER stress. (**A**) Mean fluorescence intensity values in BODIPY-stained 1-day, 5-day and 10-day old transgenic animals (WT or *paqr-1* mutant background) without (white circles) or with tunicamycin (5 μg/ml, 24 h; grey circles) treatment. Values represent means ± SEM from experiments performed in at least three separate replicates. *P < 0.05, **P < 0.01, ***P < 0.001. (**B**) Confocal images of day 4 strain VS29 (GFP::DGAT-2) animals in the presence of EV or *paqr-1(RNAi)* without (control) or with tunicamycin (5 μg/ml, 24 h) treatment. Representative images are shown (scale bar, 50 μm) with magnified partial frame images to clearly depict the differences in lipid droplet size.

Adiponectin receptors have been implicated in several aspects of lipid metabolism^15, 23, 24^. Notably, disruption of the UPR^ER^ in *C. elegans* causes severe defects in the intestine, the major lipid storage tissue of these animals^25^. Thus, we investigated the involvement of PAQR-1-mediated signalling on lipid metabolism under conditions inducing UPR^ER^. To this end, we measured lipid content in WT animals and *paqr-1* mutants during ageing under ER stress. one- and five-day old *paqr-1(tm3262)* animals responded to tunicamycin by decreasing their lipid content (*vs*. control), but this response was absent in ten-day old animals (Fig. 5A). The decreased fat accumulation in stressed one- and five-day old *paqr-1* mutants is not a consequence of altered food uptake or defecation rates, since these ultradian rhythms were not affected compared to WT animals (Supplementary Fig. S6). Importantly, loss of lipid catabolic responses upon ER stress in ten day-old *paqr-1(tm3262)* mutants (Fig. 5A) coincides with loss of the capacity to activate UPR^ER^ at this age (Fig. 2C), indicating that a UPR^ER^-mediated mechanisms might be involved in lipid clearance observed in PAQR-1-depleted animals. Given that adiponectin signalling suppresses lipolysis in mouse adipocytes^24^, we investigated whether impairment of PAQR-1 similarly induces lipid droplet clearance. We examined the consequences of *paqr*-1 silencing in WT *C. elegans* animals on lipid droplet size under ER stress conditions. The presence of tunicamycin significantly decreased droplet size upon silencing of *paqr-1* (Fig. 5B and Supplementary Fig. S9). These findings indicate that both PAQR-1 and UPR^ER^ modulate lipid droplet metabolism.

**Figure 6:**
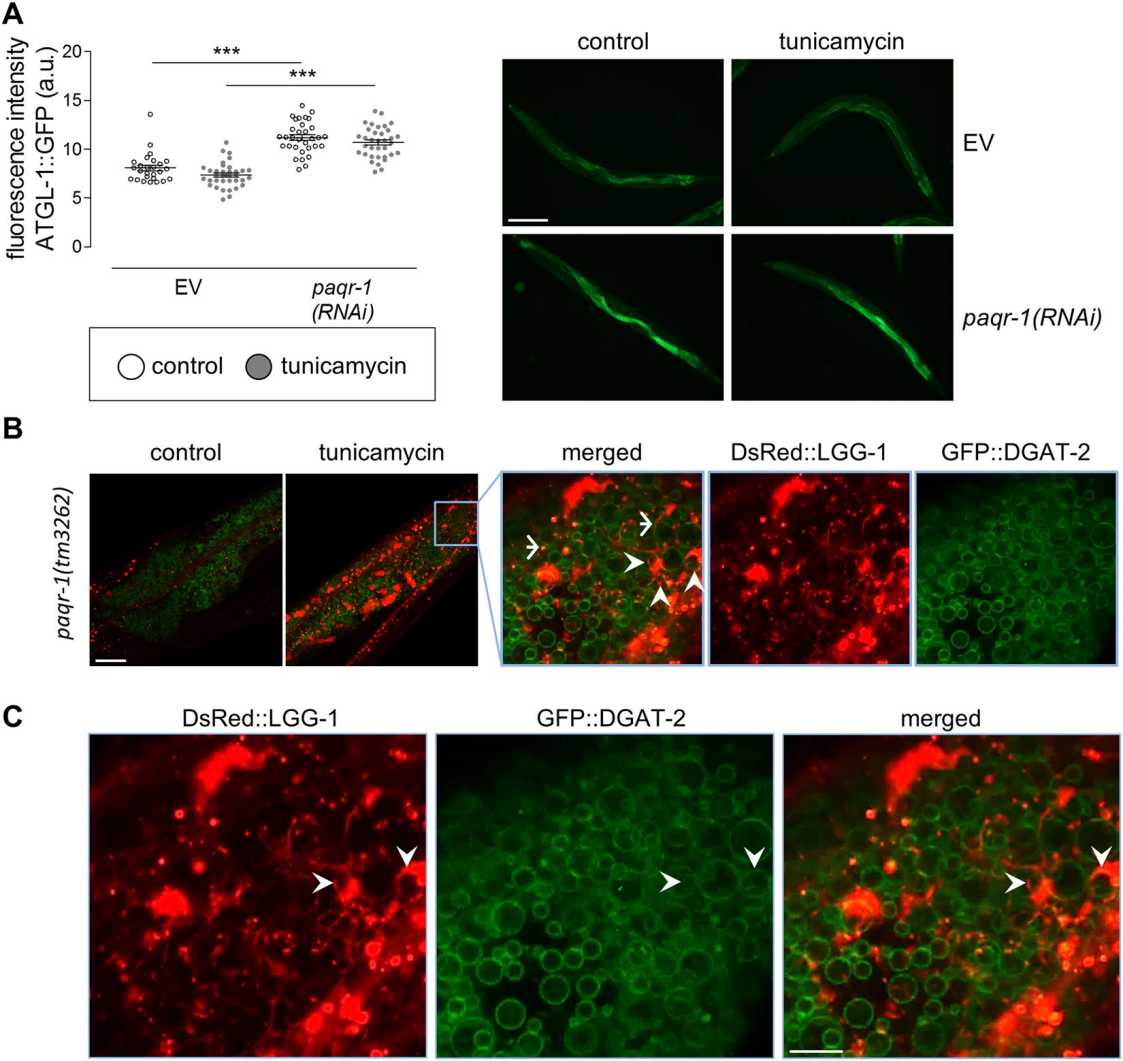
PAQR-1 appears to regulate ATGL-1 expression independently of ER stress, which induces selective autophagy. (**A**) Mean fluorescence intensity values in day 4 VS20 (ATGL-1::GFP) animals in the presence of EV or *paqr-1(RNAi)* and without (white) or with 5 μg/ml tunicamycin (grey). Representative images are shown. Scale bar, 100 μm. Values represent means ± SEM from experiments performed on at least three separate occasions. ***P < 0.001. (**B**) Induction of lipid droplet-specific autophagy is signified by co-localization of GFP (GFP::DGAT-2) and DsRed (DsRed::LGG-1). Representative confocal images are shown (scale bar, 20 μm): arrowheads indicate lipid droplet surfaces (green) surrounded by autophagosomal protein LGG-1 (red), and arrows indicate co-localization of LGG-1-containing punctae (red) and lipid droplets (green). (**C**) Enlarged images of panel B are shown (scale bar, 4 μm). Arrowheads indicate lipid droplet surfaces (green) surrounded by autophagosomal protein LGG-1 (red).

Next we investigated the mechanisms by which *paqr-1* knockdown induces lipid catabolism. Lipolysis is defined as the hydrolysis of triacylglycerols (TGs) stored in cellular lipid droplets. The adipose triglyceride lipase (ATGL/ATGL-1) catalyses the initial hydrolysis step of the vast majority of TGs. Adiponectin binding to adiponectin receptors stimulates activation of AMP-activated protein kinase (AMPK) and the proliferator-activated receptor alpha (PPARα)^14^. In *C. elegans* the homolog of AMPK has been shown to inhibit *atgl-1* ^26^. We find that tunicamycin does not affect ATGL-1 expression in control animals, whereas *paqr-1*(*RNAi*) appears to increase ATGL-1 protein expression under both control and ER stress conditions, to similar levels, without altering autofluorescence (Fig. 6A and Supplementary Fig. S10). These observations suggest that in addition to the ATGL-1 lipase, other mechanisms mediate lipid droplet clearance observed during ER stress and ageing, upon PAQR-1 depletion.

A selective type of autophagy, specific for lipid droplets (also referred to as lipophagy) has been reported^27–29^. We examined the contribution of lipophagy on lipid droplet clearance, in PAQR-1 deficient animals. We developed a platform that allows monitoring of lipophagy *in vivo* by simultaneously expressing the lipid droplet-localized protein DGAT-2 fused with GFP and the autophagy specific protein LC3/LGG-1 fused with DsRed, in nematode cells and tissues of interest. Co-localization of these two proteins signifies induction of lipophagy. Using this system, we find that tunicamycin induces expression of LC3/LGG-1, confirming that general autophagy is elevated under ER stress. Notably, LC3/LGG-1 is localized in structures where DGAT-2 is also localized (Fig. 6B). Indeed, DGAT-2-positive lipid droplets were often fully surrounded by LC3/LGG-1 (Fig. 6B), indicating that selective lipophagy mediates lipid droplet clearance. These findings indicate that PAQR-1 adiponectin receptor signalling modulates lipid metabolism via lipophagy, under conditions of ER stress and during ageing.

### Autophagy is required for UPR^ER^ activation while it is dispensable for enhanced survival upon PAQR-1 depletion during ER stress

**Figure 7:**
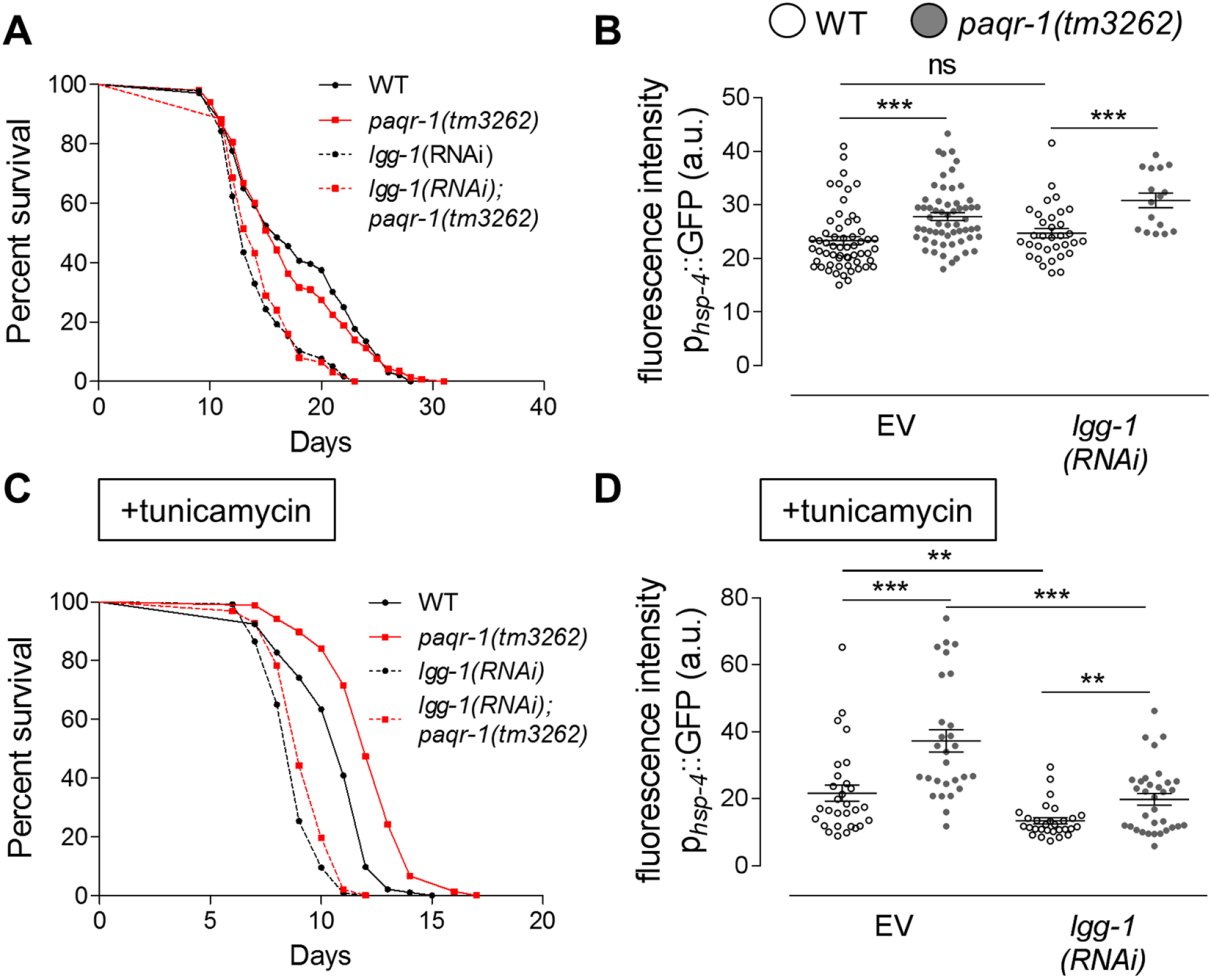
Inhibition of autophagy does not perturb PAQR-1-dependent survival but compromises UPR^ER^ activation during ER stress. (**A**) Survival curves of WT (black) and *paqr-1(tm3262)* mutant (red) animals subjected to *lgg-1(RNAi)* (dashed) under normal conditions. Lifespan values are given in Table S1. (**B**) Mean fluorescence intensity values of day 4 p_*hsp-4*_GFP transgenic animals (WT (white) or *paqr-1* mutant (grey) background) in the presence of EV or *lgg-1(RNAi)*. (**C**) Survival curves of WT (black) and *paqr-1(tm3262)* mutant (red) animals subjected to *lgg-1(RNAi)* (dashed) and under ER stress (tunicamycin, 50 μg/ml). Lifespan values are given in Table S1. (**D**) Mean fluorescence intensity values of day 4 p_*hsp-4*_GFP transgenic animals (WT (white) or *paqr-1* mutant (grey) background) in the presence of EV or *lgg-1(RNAi)* and tunicamycin (50 μg/ml, 24 h). Values in histograms represent means ± SEM from experiments performed on at least three separate occasions. **P < 0.01; ***P < 0.001.

Given that PAQR-1 depletion promotes ER stress-dependent survival and concomitant lipid metabolism through induction of lipophagy, we examined the requirement for autophagy in survival upon ER stress. We silenced two key autophagy components, LC3/LGG-1 and Beclin1/BEC-1, in WT and *paqr-1(tm3262)* genetic backgrounds and assayed for lifespan and UPR^ER^ induction (Fig. 7, for *lgg-1(RNAi)*; Supplementary Fig. S11, for *bec-1(RNAi)*). LGG-1 or BEC-1 deficiency reduced survival under normal and ER stress conditions both in WT animals and *paqr-1(tm3262)* mutants. Under normal conditions, UPR^ER^ induction monitored by *p*_*hsp-4*_GFP, was not much affected by LGG-1 or BEC-1 silencing in either WT animals or *paqr-1(tm3262)* mutants. However, LGG-1 or BEC-1 deficiency decreased *p*_*hsp-4*_GFP expression under ER stress in both WT and *paqr-1(tm3262)* animals. By contrast, autophagy is not required for UPR^ER^ activation upon PAQR-1 depletion. These findings, coupled with observations described above, indicate a reciprocal, feed-forward loop between ER stress and autophagy, with ER stress promoting autophagy and autophagy promoting UPR^ER^. Nevertheless, mitigation of autophagy is largely not mediating *paqr-1(tm3262)*-dependent effects on UPR^ER^ activation.

## Discussion

Our study directly implicates adiponectin signalling in the maintenance of proteostasis under conditions of stress, and the regulation of lipid metabolism during ageing. Understanding how distinct pathways coordinate to influence metabolism and stress resistance across different tissues at the organismal level is a critical focal point of ageing research. Adipokines are prospective mediators of intercellular and inter-tissue communication by acting both locally, in an autocrine or paracrine fashion, or systemically in an endocrine fashion^14^. Thus, the adipose tissue can communicate with distant organs and the central nervous system (CNS) bidirectionally, to modulate the ageing process. We find that PAQR-1, the closest *C. elegans* homolog of human adiponectin receptor 1 (AdipoR1), is an important determinant of ER stress resistance and survival under conditions of stress. Notably, the effects of PAQR-1 on ER stress resistance and survival under stress oppose to those exerted by the two other adiponectin receptors, PAQR-2 and PAQR-3, in *C. elegans*. Interestingly studies on energy metabolism in adiponectin receptor knock-out (KO) mice have also revealed opposing effects of AdipoR1 and AdipoR2^30^.

Our findings indicate that PAQR-1 plays an important role in reprogramming ER homeostasis. *paqr-1(tm3262)* mutant animals exhibited elevated basal expression of the GRP78/BiP homolog, *hsp-4*, and enhanced expression under ER stress, suggesting that PAQR-1 functions to negatively regulate UPR^ER^. Exposure to mild ER stress has been shown to have a protective “preconditioning” effect that enables cells to better adapt to severe stress stimuli. This phenomenon, termed hormesis, has been described in various organisms including *C. elegans* and has attracted much attention, given that slight UPR^ER^ activation may represent an effective strategy to alleviate age-associated diseases and augment lifespan^31–34^. For example, hormesis has been shown to protect brain and heart cells under ischemia^35, 36^, and to ameliorate neurodegeneration in Drosophila and mouse models of Parkinson’s disease, via mechanisms involving an interplay between ER stress responses and autophagy^32, 37^. Numerous additional examples of ER hormesis, relevant to a variety of human diseases have been described, raising the possibility that hormesis can be exploited for therapeutic interventions^38^. We found that basal UPR^ER^ activation is elevated in animals lacking functional PAQR-1. Therefore, these mutants are experiencing mild ER stress, which hormetically enhances responsiveness to acute ER stress and promotes survival. Basal UPR^ER^ induction requires activation of the SKN-1 transcription factor.

ER stress is a potent inducer of autophagy, and autophagy has been linked to ER stress resistance^39^. *C. elegans* mutants lacking HPL-2, the homolog of heterochromatin protein 1 (HP1), show elevated autophagic flux following ER stress, and increased survival, which has been attributed to hormesis^31^. We found that abrogation of general autophagy by *lgg-1(RNAi)* markedly reduces UPR^ER^ activation and survival following ER stress, but does not alleviate the effects of PAQR-1 depletion. This suggests that elevated basal UPR^ER^ in *paqr-1(tm3262)* mutants is sufficient to engage hormetic protection. However, the beneficial effects of *paqr-1* lesions on ER stress resistance and survival decreased during ageing, in association with deterioration of UPR^ER^, indicating that inactivation of PAQR-1 *per se* is insufficient to reverse the detrimental consequences of ageing when UPR^ER^ is already impaired. In this context, ectopic expression of the spliced form of XBP-1 in aged *C. elegans* was shown to constitutively activate the UPR^ER^, increase resistance to ER stress and prolong animal lifespan^17^.

While adiponectin signalling has been postulated to play a role in age-associated pathology, there is limited information directly linking adiponectin to ageing and the regulation of longevity. The lifespan of high-fat diet-fed AdipoR1-deficient and AdipoR2-deficient mice, as well as AdipoR1/AdipoR2-double knockout mice was found to be shorter, compared to wild type^40^. In *C. elegans*, we find that although loss of PAQR-1 depletion does not alter lifespan under physiological conditions, it significantly improves survival under ER stress and also enhanced longevity of animals with disrupted insulin/IGF-1 signalling. These findings indicate that the *C. elegans* PAQR-1 receptor is a negative regulator of longevity under conditions of ER stress.

We find that both ER stress-activated transcription factors (XBP-1, ATF-4) and general stress defence transcription factors (SKN-1 and to a lesser extend DAF-16) mediate *paqr-1* lesion effects on lifespan, UPR^ER^ and survival. Although DAF-16 and SKN-1 both influence ageing and are inhibited by the insulin/IGF-1 pathway, their requirement with respect to longevity and stress responses differs^21^. Indeed, the impact of SKN-1 and/or DAF-16 silencing on stress resistance and survival upon *paqr-1* lesion varied according to growth conditions (normal vs. ER stress). This indicates that differential transcription regulation by DAF-16 and SKN-1 serves to fine tune proteostasis^41^. Notably, DAF-16 and SKN-1 function varies depending on tissue/cell type and genetic background^20, 42–44^. Cell non-autonomous UPR^ER^ activation plays an important role in stress resistance and ageing^17, 45^. DAF-16 predominantly exerts its effects in the nervous system and intestinal cells, whereas SKN-1 is constitutively localized to ASI neuron nuclei and accumulates in intestinal nuclei in *daf-2* mutant animals. Thus, DAF-16, SKN-1 and the ER specific transcription factors regulate both distinct and overlapping sets of genes, in multiple tissues, depending on, environmental cues, physiological and genetic background, to modulate ageing.

Although, autophagy has been implicated in lipid storage and turnover^46^, the impact of this association on lifespan is not fully understood. Indeed, the role of the related, selective type of autophagy, lipophagy, the potential involvement of adiponectin receptors is unknown. The intestine, which is the main metabolic tissue and serves as the main site for lipid storage in *C. elegans*, is the tissue most responsive to ER stress and it is highly sensitive to ER proteostasis-perturbations^17, 25, 47^. Our findings indicate an association between UPR^ER^ and lipid metabolism. This association is dependent on PAQR-1 and selective lipophagy. We find that induction of lipid catabolism and lower body fat enhances survival under conditions of ER stress. Notably, accumulating evidence link chronic ER stress and UPR^ER^ defects to lipotoxicity, manifested as cellular abnormalities and cell death due to excessive lipid accumulation in non-adipose tissue. We hypothesize that a hormetic response triggered by loss of PAQR-1 protects from lipotoxicity, promoting longevity. Adiponectin KO mice show reduced liver lipid accumulation when fed a high-fat diet, with the effects attributed partially to the down-regulation of lipogenic genes^23^. In *C. elegans*, we find that PAQR-1 regulates expression of the lipolytic enzyme ATGL, suggesting that adiponectin signalling balances lipolytic and lipogenic pathways. PAQR-1 depletion appears to increase ATGL-1 expression, and reduces lipid content and droplet size, indicating that PAQR-1 is a central mediator of fat accretion by serving as a relay node for nutrient sensing pathways.

In addition to its insulin sensitizing function, adiponectin has a central role in energy homeostasis, and has been proposed to serve as a starvation signal invoked upon fat reserve insufficiency. In turn, adiponectin promotes nutrient intake, decreases energy expenditure and promotes fat accumulation. Our findings show that disruption of adiponectin signalling mimics perturbed or inadequate nutrient intake, which, coupled with UPR^ER^, triggers catabolic processes such as autophagy, including selective ER-phagy and lipophagy, to access and mobilize internal nutrient stores. Thus adiponectin receptor inactivation may represent a mechanism to promote survival and longevity under conditions of stress. Our findings further delineate an important role for PAQR-1, the *C. elegans* homolog of AdipoR1, in the regulation of metazoan lifespan under stress conditions, by linking cellular metabolism and stress responses. Combined, these observations indicate that impaired adiponectin signalling may underlie reduced nutrient sensing and metabolic adaptation during ageing and stress, leading to a range of age- and obesity-associated pathologies in humans.

## Materials and Methods

### Strains

Animals were maintained under standard culture conditions. The following strains were used in this study. The wild-type (WT) strain N2 (Bristol) was used as the reference strain. Other strains also used: QC128: *paqr-1(tm3262) IV*, QC129: *paqr-2(tm3410) III*, QC130: *paqr-3(ok2229) IV*, TM2457: *xbp-1(tm2457)* III, RB772: *atf-6(ok551) X*, RB545: *pek-1(ok275) X*, SJ4005: *N2;Is[p*_*hsp4*_GFP*] V*, VS29: N2;Is[p_*vha-6*_3xFLAG::TEV::GFP::DGAT-2], VS20: N2;Is[p_*atgl-1*_ATGL-1::GFP + mec-7::RFP], QC128xVS29;Ex[p_*lgg-1*_DsRed::LGG-1]. To construct double mutants, *paqr-1* males were mated to hermaphrodites carrying the mutation of interest, and the presence of the respective mutations was checked by RT-PCR using the primers listed below. For *atf-6(ok551)*: 5′-AATGACCAGGAAATGTGGGA-3′ and 5′-AAGTGTCAATTGGCCAGTCC-3′, for *pek-1(ok275)*:5′-CTGAGAAGGCAACGCTCTCT-3′ and 5′-ATCACCGCTACTCTGGATGG-3′, for *xbp-1(tm2457)*: 5′-CAACAATCGCAGAAGCAGAA-3′ and 5′-AACCGGGAGGAGAATAGGG-3′, for *paqr-1(tm3262):* 5′-AGGGCAATGGCGTGAAATAAAC-3′ and 5′-AACGAGAGTCCAAGACATAGAACGG-3′.

### RNAi

For RNAi experiments, worms were placed on NGM plates containing 2 mM IPTG and seeded with HT115(DE3) bacteria transformed with either the pL4440 vector (EV: empty vector control) or the test RNAi construct. For engineering the *paqr-1* RNAi construct, gene specific region of interest was obtained by PCR amplification from *C. elegans* genomic DNA using the following set of primers 5′-TCTAGAATGAATCCAGATGAGGTCAATCG-3′ and 5′-AACGAGAGTCCAAGACATAGAACGG-3′. The fragment generated was subcloned into the pL4440 plasmid vector and generated vector was transformed into HT115(DE3). Efficiency of silencing was determined by qPCR using the following set of primer 5′-ACAGCACAACTGTACAGGTGAAA-3′ and 5′-TCCCTTTTTACGACGATATCTAAGA-3′. The rest RNAi constructs (*daf-2(RNAi), skn-1(RNAi), daf-16(RNAi), lgg-1(RNAi), bec-1(RNAi)*) that were used have been previously described^48^.

### Lifespan analysis

Lifespan analyses were performed at 20 °C, unless otherwise indicated. Eighty to one hundred and fifty synchronous animals were scored per condition. Lifespans were performed on *E. coli* OP50 or HT115. For lifespan assays that involved RNA silencing, worms were exposed to RNAi from the L4 stage. Animals were transferred to fresh plates every 2–4 days thereafter and examined every day for touch-provoked movement and pharyngeal pumping, until death. Worms that died owing to internally hatched eggs, an extruded gonad or desiccation due to crawling on the edge of the plates were censored and incorporated as such into the data set. Each survival assay was repeated at least twice and figures represent typical assays. Survival curves were created using the product-limit method of Kaplan and Meier.

### ER Stress resistance assays

Gravid adult WT or mutant worms were placed on nematode growth medium (NGM) plates, seeded with OP50 or HT115 bacteria, containing tunicamycin (5 μg/ml) or trapsigargin (15 μM) (Sigma-Aldrich). Control animals were incubated in an equivalent dilution of DMSO in M9. Animals were allowed to lay eggs for up to 4h. Total number of eggs was counted and compared with the number of animals that reached the L4/adult stage within 72 to 96 h at 20 °C. For each strain approximately 100–200 eggs were placed on each of four independent plates.

### Heat stress survival assays

To evaluate thermotolerance, adult WT or mutant animals were placed on prewarmed (37 °C) NGM plates and incubated at 37 °C. Worms were scored for motility and provoked movement after 4, 6, 9 and 11 h. Worms failing to display these traits were scored as dead.

### Lipid droplet staining

For the staining of *C. elegans* lipid droplets a fluorescence-based fixative method was applied. Adult WT or mutant animals were washed three times in M9 buffer and fixed with 4% paraformaldehyde for 10 min at RT, followed by two freeze thaw cycles (5 minutes at −80 °C and 5 minutes at 40 °C). Worms were again washed three times and stained with C1-*BODIPY*-C12 500/510 (D3823) (1 µg/ml) (Thermos Scientific, Cat# D3823) for one hour at RT. Worms were washed and mounted on coverslips for microscopic examination.

### Microscopy and quantification

Worms were immobilized with levamisole before mounting on coverslips for microscopic examination with a Zeiss AxioImager Z2 epifluorescence microscope or a Zeiss LSM 710 mutliphoton confocal microscope. Images were acquired under the same exposure conditions. Average pixel intensity values were calculated by sampling images of different animals. We calculated the mean pixel intensity for each animal in these images using the ImageJ software (http://rsb.info.nih.gov/ij/). For each experiment, at least 40 animals were examined for each strain/condition and each assay was repeated at least three times. For the quantification of lipid droplets, the ImageJ software was used to assess droplet surface. A 365/420 nm filter set was utilized for the detection of the lipofuscin signal. A Zeiss SteREO Lumar V12 Stereo Microscope was also used to acquire plate images.

### RT-PCR and qPCR analyses

Total RNA was harvested from synchronous populations, using TRIzol (Invitrogen) for lysing. For cDNA synthesis, mRNA was reverse transcribed using a iScriptTM cDNA Synthesis Kit (BioRad) and PrimeScriptTM Reverse Transcriptase (Takara). Quantitative PCR was performed in triplicate using a Bio-Rad CFX96 Real-Time PCR system (Bio-Rad). The following set of primers was used for paqr-1: 5′-ACAGCACAACTGTACAGGTGAAA-3′ and 5′-TCCCTTTTTACGACGATATCTAAGA-3′ and results were normalized to genomic DNA using the following primers for pmp-3: 5′-ATGATAAATCAGCGTCCCGAC-3′ and 5′-TTGCAACGAGAGCAACTGAAC-3′.

### Ultradian rhythm monitoring

Defecation cycles of synchronized animals were scored under a stereomicroscope. For each strain at least 30 animals were scored for three cycles each. Pharyngeal pumping rate was scored by measuring grinder movements under a stereomicroscope. Approximately 30 synchronized animals were monitored and grinder movement over a period of 20 seconds were counted. The feeding rate of each animal was calculated by averaging the three measures from each animal (pumps per 20 s) and subsequently by multiplying by 3.

### Statistical analysis

All experimental series were repeated independently at least three times. Data are given as mean ± SEM. Prism 5 (GraphPad Software) software package was used for statistical analyses. The log-rank (Mantel–Cox) test was used to evaluate differences between survivals and determine *P* values. Differences among treatment groups were determined by Student’s t test and two-way ANOVA analyses. A value of *P* < 0.05 was considered statistically significant.

## Electronic supplementary material

Supplementary Information.
