# Supplementary material for: Differential adiponectin signalling couples ER stress with lipid metabolism to modulate ageing in C. elegans
Source: Sci Rep. 2017 Jul 11;7:5115. doi: 10.1038/s41598-017-05276-2 (PMC5505976; doi:10.1038/s41598-017-05276-2)
Supplement: Supplementary file 1 — Supplementary Information. [file 41598_2017_5276_MOESM1_ESM.pdf]

Differential adiponectin signalling couples ER stress with lipid metabolism to modulate ageing in *C. elegans*

Emmanouil Kyriakakis, Nikolaos Charmpilas & Nektarios Tavernarakis

Supplementary Figures S1-S11 & Supplementary Table S1

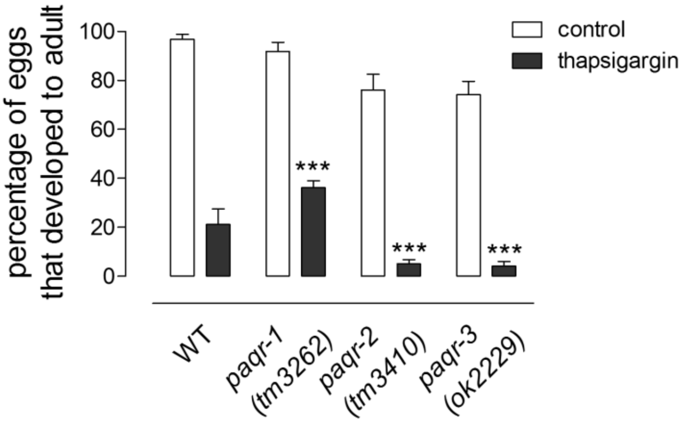

Figure S1. ***C. elegans*** **adiponectin receptor mutants respond differently to thapsigargin exposure.** Eggs from WT, *paqr-1(tm3262)*, *paqr-2(tm3410)* and *paqr-3(ok2229)* strains were laid on plates in the absence (white bars) or presence of 15 μM thapsigargin (black bars). Each strain was scored on four independent plates and each experiment was repeated independently at least three times. Error bars represent SEM for repeat plates within the experiment. Student *t* test values were calculated *vs.* WT + thapsigargin ($n \sim 150$; ***$P < 0.001$).

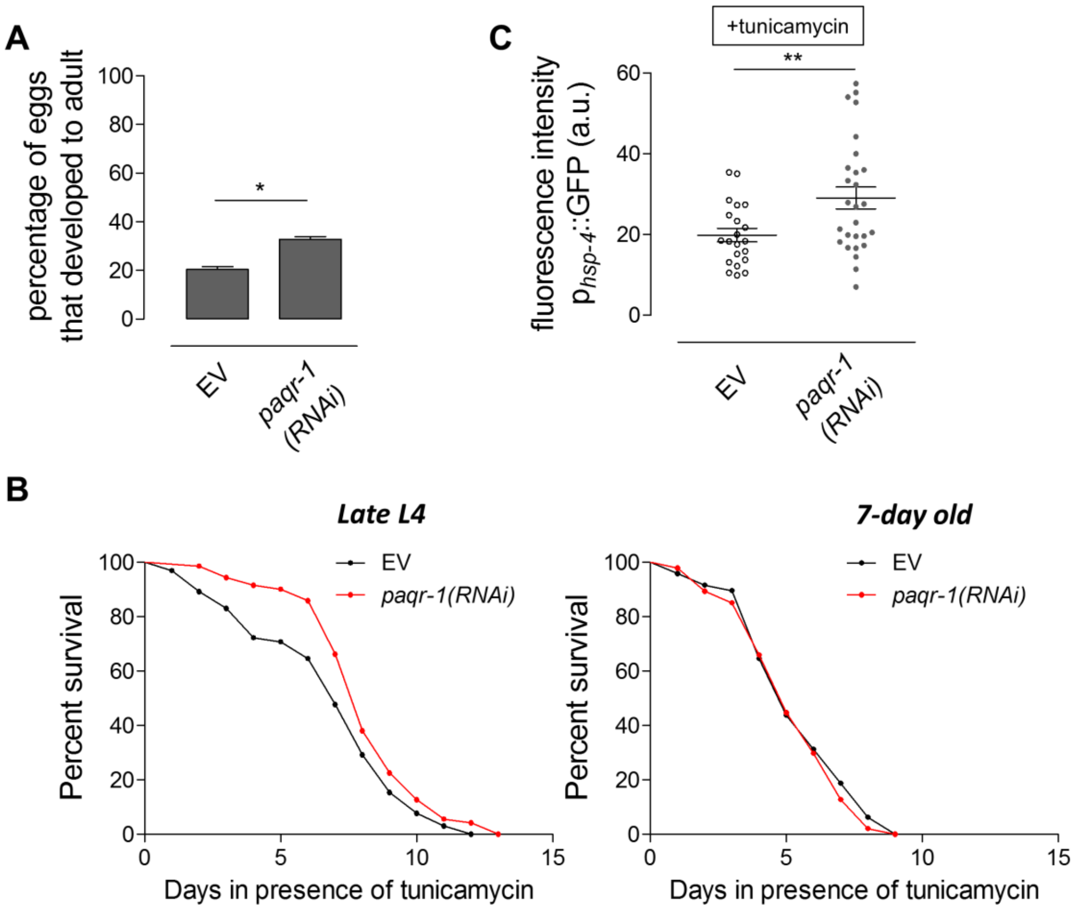

Figure S2. *paqr-1* silencing increases resistance to ER stress and augments survival of ER stressed animals. (A) Eggs from WT animals were laid on plates in the presence of 5 µg/ml tunicamycin and EV or *paqr-1(RNAi)*. Percentage of eggs that developed into mature adults was scored. (B) Survival curves of WT animals subjected to EV (black) or *paqr-1(RNAi)* (red) under ER stress (5 µg/ml tunicamycin). Lifespan values are given in Table S1. (C) Mean fluorescence intensity values of 24h tunicamycin-treated (5 µg/ml), day 4 $p_{hsp\text{-}4}$GFP transgenic animals subjected to EV (control) or *paqr-1(RNAi)* are shown. Values represent means ± SEM **P* < 0.05; ***P* < 0.01.

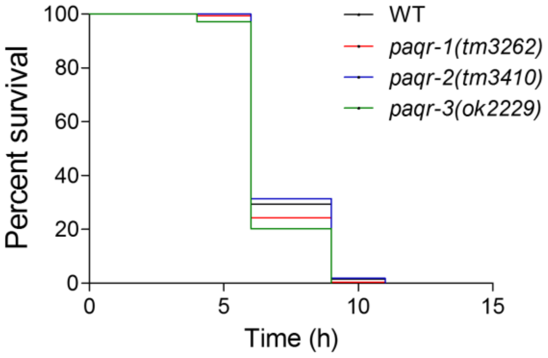

Figure S3. ***C. elegans*** **adiponectin receptor lesions do not perturb survival under heat stress conditions.** Survival of wild type and *paqr-1(tm3262), paqr-2(tm3410), paqr-3(ok2229)* mutant animals at 37 ^{o}C. The experiment was repeated independently at least three times.

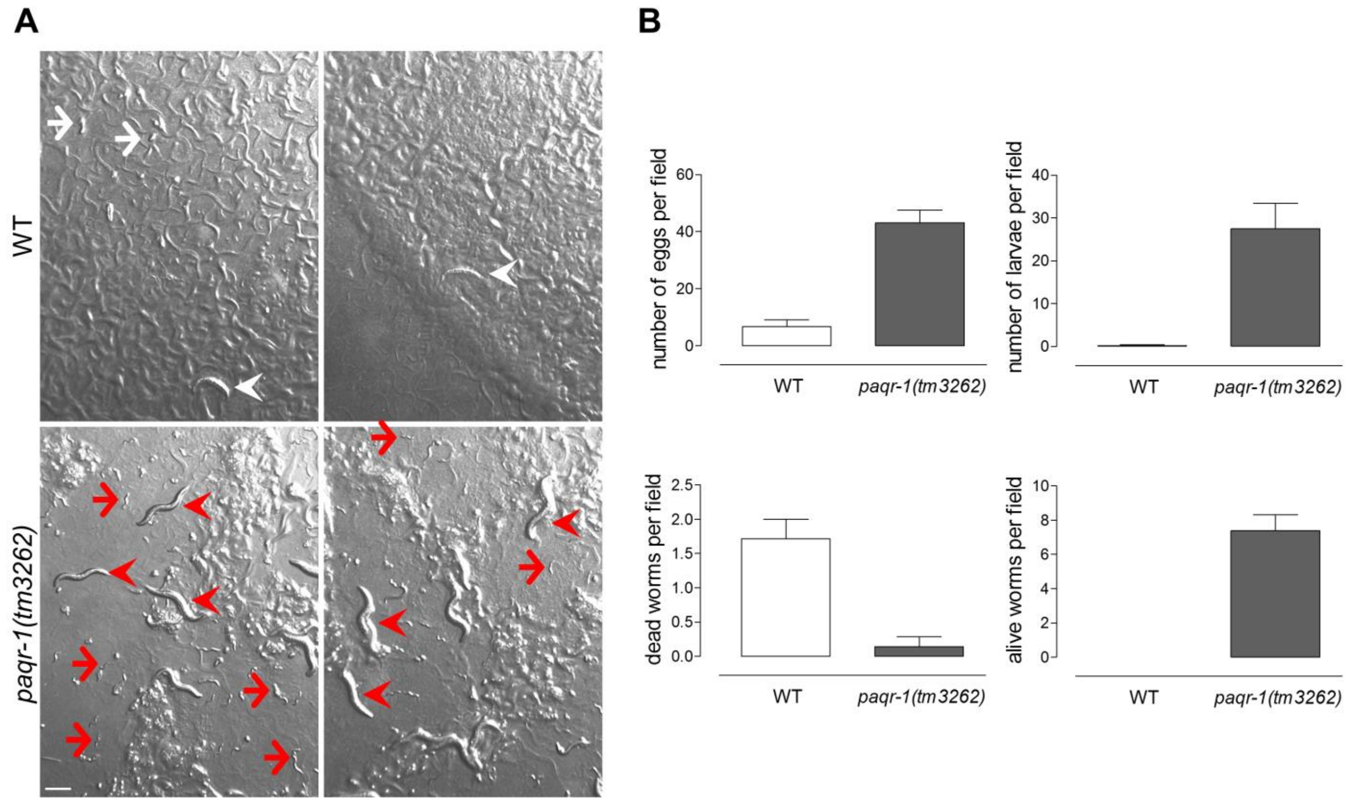

Figure S4. Tunicamycin resistance. (A) Representative images illustrating survival of WT and *paqr-1(tm3262)* strains after 8 days on tunicamycin are shown. Differences on the bacterial lawn are also evident, suggesting differences in mobility and viability. Scale bar, 250 μm. White arrowheads indicate dead animals; white arrows indicate eggs that never developed into mature adults; red arrowheads indicate live animals; red arrows indicate newly hatched eggs. (B) Quantification of survival and fertility of WT (white) and *paqr-1* mutant (grey) animals in the presence of tunicamycin. Five random field areas from two plates were analysed and results are shown as means per field.

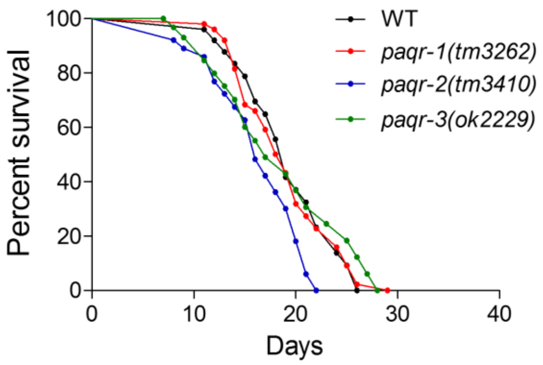

Figure S5. Longevity of adiponectin receptor mutant animals under physiological conditions. Lifespan of *paqr-1(tm3262)* and *paqr-3(ok2229)* mutant animals was comparable to wild type animals, whereas *paqr-2(tm3410)* mutant animals survived significantly less compared to wild type animals under physiological conditions. The experiment was repeated independently at least three times at 20 ^{o}C. Lifespan values are summarized in Table S1.

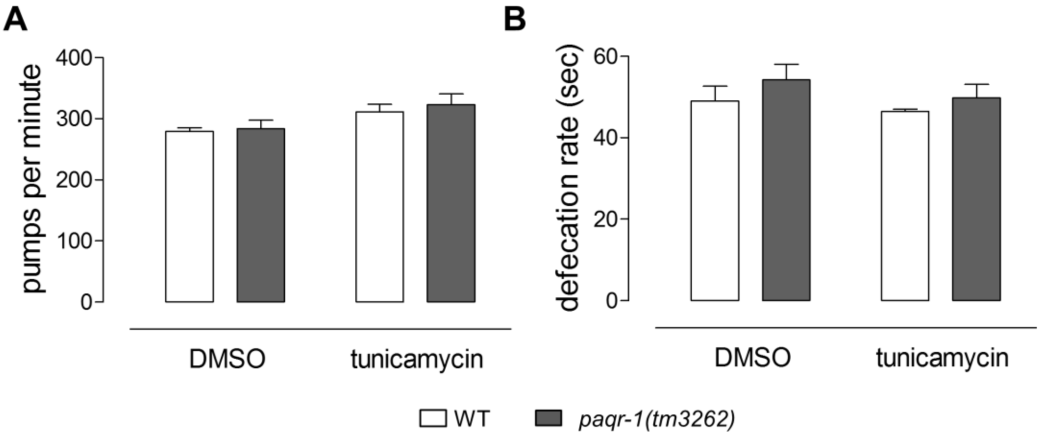

Figure S6. Ultradian rhythms are not altered by either PAQR-1 depletion or ER stress. (A) Pumping rate was not altered in *paqr-1(tm3262)*(grey bars) compared to wild type animals (white bars) in the presence or absence of 5 μg/ml tunicamycin. (B) Defecation rates were determined in the presence or absence of of 5 μg/ml tunicamycin. No significant differences were observed between *paqr-1(tm3262)* and wild type animals under these conditions. Each experiment was repeated independently at least three times.

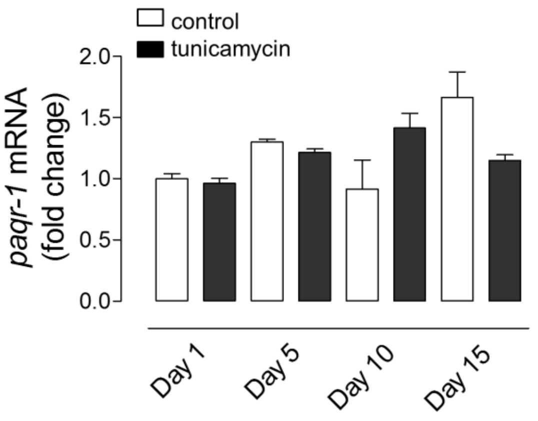

Figure S7. ER stress does not interfere with *paqr-1* mRNA levels during aging. Wild type animals were exposed to DMSO (control; white bars) or ER stress (5 μg/ml tunicamycin; black bars) and mRNA levels were calculated by qPCR during aging. No significant differences were observed. The experiment was repeated three times.

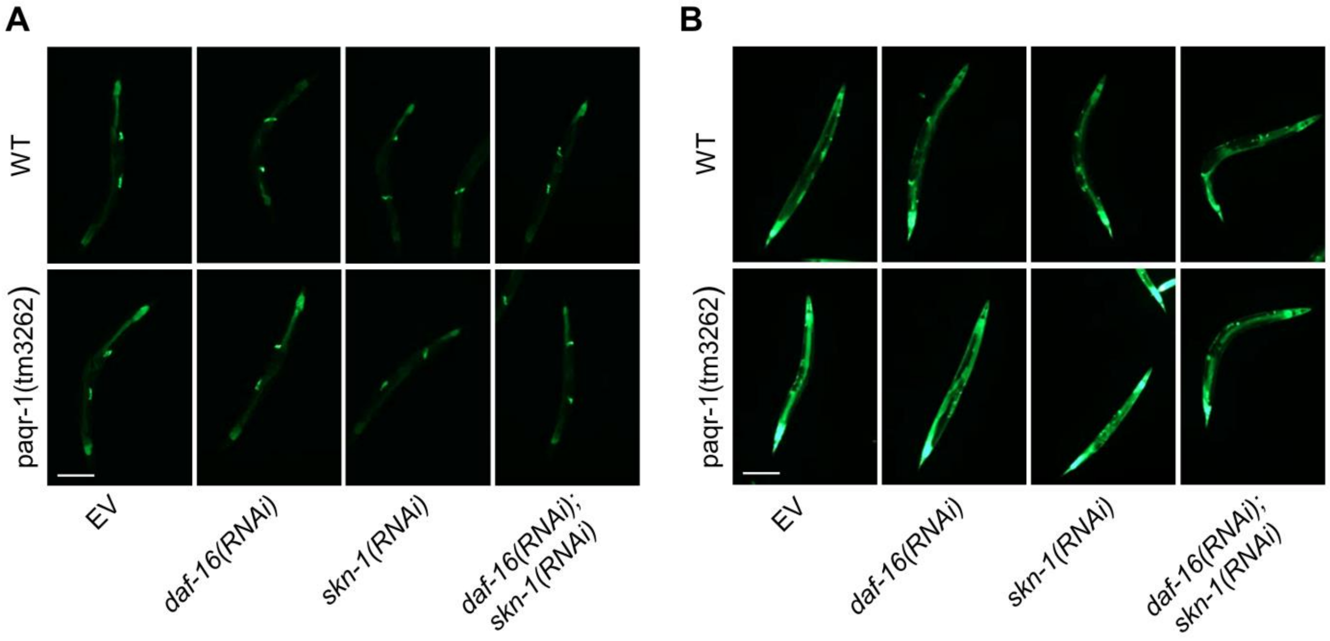

Figure S8. HSP-4 expression upon depletion of PAQR-1. HSP-4 expression levels of day-4 animals under control (A), or ER stress conditions (tunicamycin 5 μg/ml, 24h) (B). (Magnified images as shown in Fig. 4; Scale bar, 100 μm).

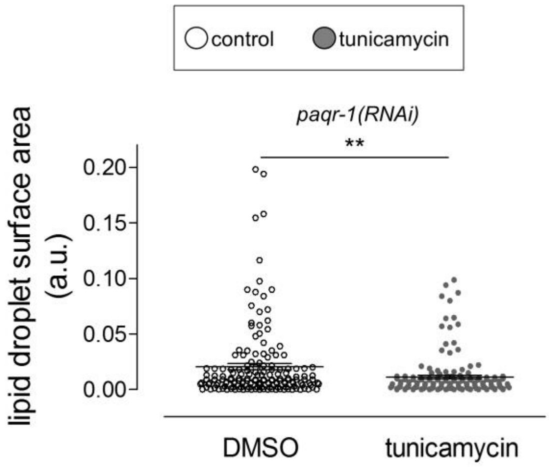

Figure S9. Quantification of lipid droplet size. Tunicamycin treatment leads to a reduction in the lipid surface area in *paqr-1(RNAi)*-treated animals. Values in histograms represent means ± SEM. **P < 0.01.

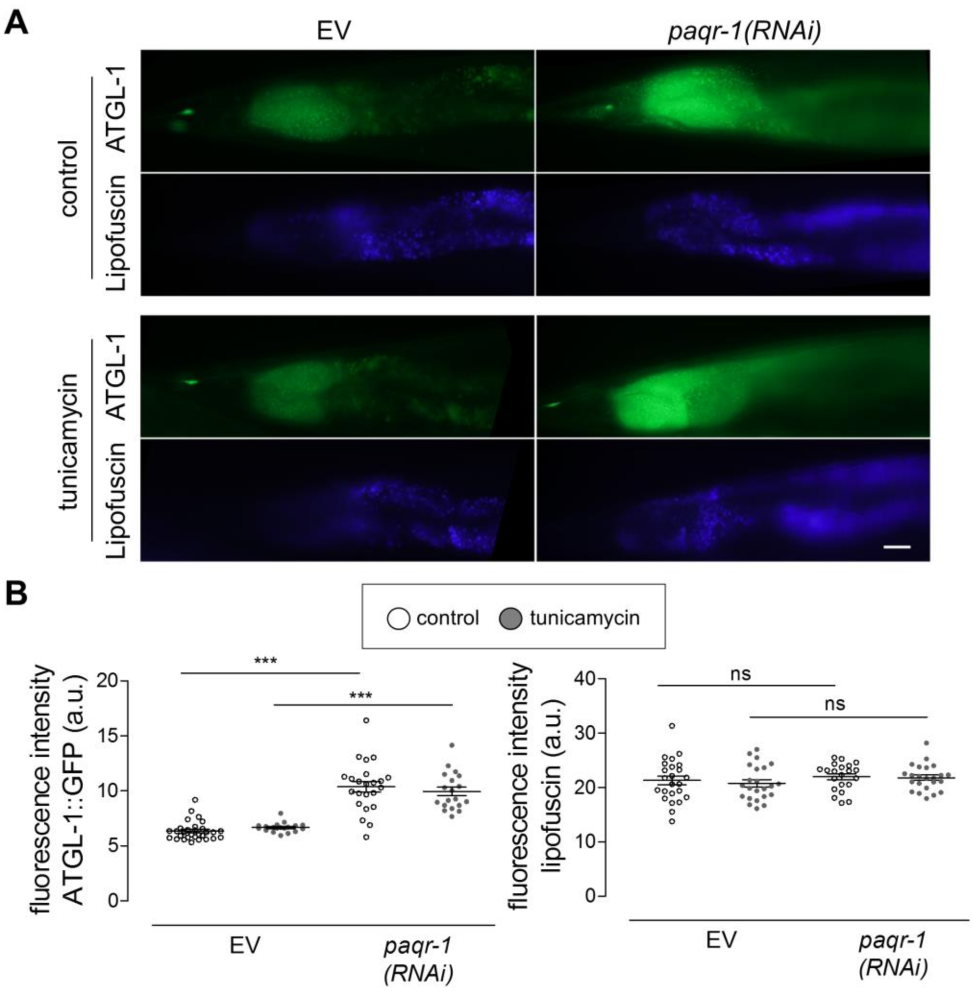

Figure S10. PAQR-1 regulates ATGL-1 expression without influencing autofluorescence. (A) Representative images for both ATGL-1::GFP (GFP filter) and autofluorescence (lipofuscin) are shown. Lipofuscin-like autofluorescence can be generated and detected across a broad spectrum[1]. To detect lipofuscin-like structures we used a 365, 420 nm filter set. GFP fluorescence was detected using a separate 470/40, 525/50 nm filter set. Scale bar, 20 µm. (B) Mean fluorescence intensity values in day 3 adult VS20 (ATGL-1::GFP) animals treated with EV or *paqr-1(RNAi),* in the presence (white) or absence of 5 µg/ml tunicamycin (grey). Mean fluorescence intensity values for lipofuscin are also quantified. Values represent means ± SEM ***P < 0.001.

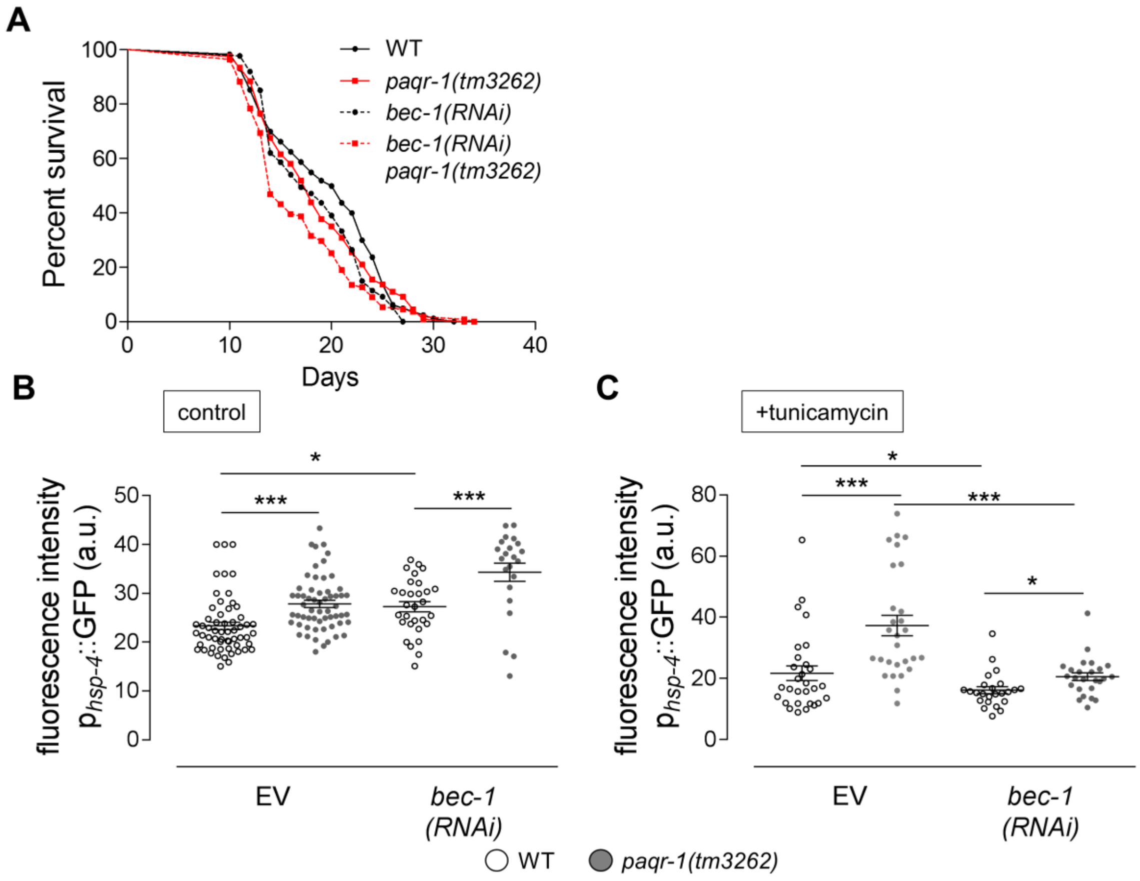

Figure S11. Silencing of *bec-1* does not affect survival of *paqr-1* mutant animals, but is required for *hsp-4* expression. (A) Survival curves of WT (black) and *paqr-1(tm3262)* mutant (red) animals treated with *bec-1(RNAi)* (dashed) under normal conditions are presented. Lifespan values are provided in Table S1. (B) Mean fluorescence intensity values of $p_{hsp\text{-}4}$GFP 4-day old transgenic animals (wild type (white) or *paqr-1* mutant (grey) background) are shown in the presence of EV or *bec-1(RNAi)*. (C) Mean fluorescence intensity values of tunicamycin-treated $p_{hsp\text{-}4}$GFP 4-day old transgenic animals (wild type (white) or *paqr-1* mutant (grey) background) are shown, in the presence of EV or *bec- 1(RNAi)*. Values in histograms represent means ± SEM from experiments performed at least trice. *P < 0.05; **P < 0.01; ***P < 0.001.

Table S1. Cumulative lifespan data. Each set of lifespan analysis is associated with its corresponding figure in the text as indicated on the left of the table. Means, standard deviation of the mean (SEM) and *P* values were calculated using the log-rank test (Mantel-Cox) from Kaplan-Meier survival analysis of cumulative data obtained from the indicated numbers of animals, as described in Materials and Methods. (SEM: standard error of the mean; tun: tunicamycin; EV: empty vector/RNAi control; ns: non-significant; WT: wild type).

	Genotype	Treatment	Mean ±SEM (days)	Deaths/Censored	*P* value
Figure 1	Wild Type (N2)	tun	8.6 ± 0.6	152/13	
	paqr-1(tm3262)	tun	11.7 ± 0.7	184/20	< 0.0001 vs. WT
	paqr-2(tm3410)	tun	3.7 ± 0.9	151/33	< 0.0001 vs. WT
	paqr-3(ok2229)	tun	7.0 ± 1.2	146/37	ns vs. WT
	Wild Type (N2)	tun (from day 7)	6.2 ± 0.3	144/28	
	paqr-1(tm3262)	tun (from day 7)	5.8 ± 0.5	138/25	ns vs. WT
	paqr-2(tm3410)	tun (from day 7)	3.3 ± 0.7	128/43	0.0003 vs. WT
	paqr-3(ok2229)	tun (from day 7)	5.5 ± 0.7	126/49	ns vs. WT
Figure 3	Wild Type (N2)	*daf-2(RNAi)*	25.5 ± 1.5	160/19	
	paqr-1(tm3262)	*daf-2(RNAi)*	39.5 ± 2.5	173/20	0.0010 vs. WT
Figure 4	Wild Type (N2)	EV	16.7 ± 0.3	150/17	
	paqr-1(tm3262)	EV	16.3 ± 0.7	149/12	ns vs. WT
	Wild Type (N2)	*daf-16(RNAi)*	12.7 ± 0.7	132/23	< 0.0001 vs. WT
	paqr-1(tm3262)	*daf-16(RNAi)*	12.2 ± 1.4	112/24	ns vs. *daf-16(RNAi)*
	Wild Type (N2)	EV	20.2 ± 0.3	166/19	
	paqr-1(tm3262)	EV	18.3 ± 0.7	175/15	ns vs. WT
	Wild Type (N2)	*skn-1(RNAi)*	17.7 ± 0.7	145/17	< 0.0001 vs. WT
	paqr-1(tm3262)	*skn-1(RNAi)*	14.0 ± 1.2	154/15	< 0.0001 vs. WT and *skn-1(RNAi)*
	Wild Type (N2)	EV + tun	11.2 ± 0.3	193/15	
	paqr-1(tm3262)	EV + tun	12.8 ± 0.7	184/14	< 0.0001 vs. WT
	Wild Type (N2)	*daf-16(RNAi)* + tun	10.0 ± 0.5	129/21	0.0002 vs. WT
	paqr-1(tm3262)	*daf-16(RNAi)* + tun	11.8 ± 0.5	155/19	ns vs. WT; 0.093 vs. *daf-16(RNAi)* + tun
	Wild Type (N2)	EV + tun	11.2 ± 0.3	193/15	
	paqr-1(tm3262)	EV + tun	12.8 ± 0.7	184/14	< 0.0001 vs. WT
	Wild Type (N2)	*skn-1(RNAi)* + tun	9.0 ± 0.5	154/10	< 0.0001 vs. WT
	paqr-1(tm3262)	*skn-1(RNAi)* + tun	12.5 ± 0.5	172/18	0.0002 vs. WT; < 0.0001 vs. *skn-1(RNAi)* + tun
Figure 7	Wild Type (N2)	EV	16.7 ± 0.3	150/17	
	paqr-1(tm3262)	EV	16.3 ± 0.7	149/12	ns vs. WT
	Wild Type (N2)	*lgg-1(RNAi)*	13.8 ± 0.7	163/6	< 0.0001 vs. WT
	paqr-1(tm3262)	*lgg-1(RNAi)*	14.3 ± 0.7	143/13	< 0.0001 vs. WT; ns vs. *lgg-1(RNAi)*
	Wild Type (N2)	EV + tun	11.2 ± 0.3	193/15	
	paqr-1(tm3262)	EV + tun	12.8 ± 0.7	184/14	< 0.0001 vs. WT
	Wild Type (N2)	*lgg-1(RNAi)* + tun	9.0 ± 0.5	156/19	< 0.0001 vs. WT
	paqr-1(tm3262)	*lgg-1(RNAi)* + tun	9.5 ± 0.5	176/20	< 0.0001 vs. WT; 0.0025 vs. *lgg-1(RNAi)*
Figure S2	EV	tun	7.0	65/7	
	paqr-1(RNAi)	tun	8.0	71/5	< 0.02 vs. EV
	EV	tun (from day 7)	5.0	68/14	
	paqr-1(RNAi)	tun (from day 7)	5.0	67/18	ns vs. EV

Figure S5	Wild Type (N2)	DMSO	19.8 ± 0.7	166/9	
	paqr-1(tm3262)	DMSO	19.3 ± 0.3	185/18	ns vs. WT
	paqr-2(tm3410)	DMSO	16.2 ± 0.2	133/29	0.0025 vs. WT
	paqr-3(ok2229)	DMSO	17.8 ± 0.7	127/45	ns vs. WT
Figure S11	Wild Type (N2)	EV	20.2 ± 0.3	166/19	
	paqr-1(tm3262)	EV	18.3 ± 0.7	175/15	ns vs. WT
	Wild Type (N2)	*bec-1(RNAi)*	18.0 ± 1.0	115/23	ns vs. WT
	paqr-1(tm3262	*bec-1(RNAi)*	15.5 ± 1.5	121/21	0.0016 vs. WT; ns vs. *bec-1(RNAi)*

References

1 Schnell, S. A., Staines, W. A. & Wessendorf, M. W. Reduction of lipofuscin-like autofluorescence in fluorescently labeled tissue. *J Histochem Cytochem* **47**, 719-730, doi:10.1177/002215549904700601 (1999).
